# Supplementary material for: Chronic trace metals effects of mine tailings on estuarine assemblages revealed by environmental DNA
Source: PeerJ. 2019 Nov 7;7:e8042. doi: 10.7717/peerj.8042 (PMC6842558; doi:10.7717/peerj.8042)
Supplement: Supplemental Information 1 — Note: unassigned species identities are indicated as notID. [file peerj-07-8042-s001.docx]

Table S1. List of marine eOTUs of aquatic Metazoan taxa recovered from sediment eDNA samples from the Rio Doce estuary. Note: unassigned species identities are indicated as notID.

| Phylum | eOTU | Phylum | eOTU |
| --- | --- | --- | --- |
| Annelida | Notobdella.nototheniae | Nematoda | Aphanolaimus.aquaticus |
|  | Aeolosoma.viride |  | Axonolaimus.sp..AxLaSp2 |
|  | Uncinais.uncinata |  | Paraphanolaimus.behningi |
|  | Haplotaxida_notID |  | Araeolaimida_notID |
|  | Eunicida_notID |  | Chromadorida_notID |
|  | Phyllodocida_notID |  | Desmodorida_notID |
|  | Boccardiella.hamata |  | Daptonema.sp..PFN.2007 |
|  | Scolelepis.sp..sco206 |  | Diplolaimella.dievengatensis |
|  | Spionida_notID |  | Eumonhystera.cf..vulgaris.1.JH.2014 |
|  | Cirrifera.dumosa |  | Metadesmolaimus.sp..PDL.2005 |
|  | Kata.leroda |  | Monhysteridae.environmental.sample |
|  | Proseriata_notID |  | Mononchus.aquaticus |
| Cnidaria | Actiniaria_notID |  | Theristus.acer |
|  | Zoantharia_notID |  | Monhysterida.D_9__uncultured.eukaryote |
|  | Bougainvillia.carolinensis |  | Monhysterida_notID |
|  | Moerisia.sp..AGC.2001 |  | Euteratocephalus.palustris |
|  | Anthoathecata_notID |  | Aphelenchoides.bicaudatus |
|  | Siphonophorae.D_9__ | | Tylenchida_notID |
|  | Hydroidolina_notID |  | Chromadorea_notID |
|  | Ellipsomyxa.adlardi |  | Mesodorylaimus.cf..nigritulus.AV.2005 |
|  | Bivalvulida_notID |  | Dorylaimida_notID |
|  | Craspedacusta.sowerbyi |  | Isomermis.lairdi |
|  | Limnomedusae_notID |  | Mermithida_notID |
| Bryozoa | Ctenostomatida_notID |  | Mononchida_notID |
| Arthropoda  Subphylum Crustacea | Bestiolina.similis |  | Trichocephalida_notID |
|  | Copepoda.environmental.sample |  | Adoncholaimus.sp. |
|  | Parvocalanus.crassirostris |  | Anoplostoma.sp..BHMM.2005 |
|  | Subeucalanus.pileatus |  | Oncholaimidae.sp..HCL2 |
|  | Calanoida_notID |  | Oncholaimidae.sp..MHMH.2008 |
|  | Eucyclops.macruroides |  | Rhabdolaimus.aquaticus |
|  | Cyclopoida.D_11_ | | Enoplida_notID |
|  | Cyclopoida_notID |  | Epitobrilus.stefanskii |
|  | Nitokra.spinipes |  | Prismatolaimus.cf..dolichurus.JH.2004 |
|  | Harpacticoida.__ |  | Triplonchida_notID |
|  | Poecilostomatoida_notID | Nemertea | Heteronemertea_notID |
|  | Copepoda_notID | Platyhelminthes | Catenula.sp..KL.2009 |
|  | Chrissia.dongqianhuensis |  | Catenulidae_notID |
|  | Ilyocypris.japonica |  | Rhynchoscolex.simplex |
|  | Limnocythere.inopinata |  | Stenostomum.leucops |
|  | Semicytherura.striata |  | Stenostomum.simplex |
|  | Podocopida.D_11__uncultured.eukaryote |  | Stenostomidae.D_9 |
|  | Podocopida_notID |  | Stenostomidae_notID |
| Gastrotricha | Aspidiophorus.ophiodermus |  | Dalyellioida_notID |
|  | Chaetonotidae.gen..sp..ARSG.2016 |  | Gyratrix.hermaphroditus |
|  | Chaetonotus.cf..dispar.TK146 |  | Kalyptorhynchia_notID |
|  | Chaetonotus.cf..similis.TK230 |  | Dochmiotrema.limicola |
|  | Chaetonotus.heideri |  | Promesostoma.sp..WW.2004 |
|  | Halichaetonotus.sp..4.TK.2012 |  | Strongylostoma.elongatum |
|  | Heterolepidoderma.loricatum |  | Neodalyellida_notID |
|  | Heterolepidoderma.sp..1.TK.2012 |  | Bothrioplana.sinensis |
|  | Chaetonotida_notID |  | Neocalceostomoides.spinivaginalis |
|  | Chaetonotida_notID_2 |  | Diplectanidae_notID |
|  | Chaetonotida_notID3 |  | Maricola_NotID |
|  | Redudasys.fornerise |  | Macrostomida_notID |
| Gnathostomulida | Bursovaginoidea_notID | Rotifera | Bdelloidea_notID |
| Micrognathozoa | Limnognathia.maerski |  | Collotheca.campanulata |
| Mollusca | Caenogastropoda_notID |  | Conochilus.hippocrepis |
|  | Nucula.proxima |  | Filinia.longiseta |
|  | Myoida_notID |  | Flosculariacea_notID |
|  | Corbicula.fluminea |  | Ascomorpha.ovalis |
|  | Venerida_notID |  | Ploimida_notID |
|  |  | Tardigadra | Mesobiotus.philippinicus |
|  |  |  | Parachela_notID |
