# Supplementary material for: Chronic trace metals effects of mine tailings on estuarine assemblages revealed by environmental DNA
Source: PeerJ. 2019 Nov 7;7:e8042. doi: 10.7717/peerj.8042 (PMC6842558; doi:10.7717/peerj.8042)
Supplement: Supplemental Information 2 [file peerj-07-8042-s002.docx]

Table S2. Dominant meiofaunal OTUs with > 0.1% of sequence variant reads included in multivariate analysis

| Phylum | eOTU | Phylum | eOTU |
| --- | --- | --- | --- |
| Annelida | Notobdella.nototheniae | Nematoda | Adoncholaimus.sp. |
|  | Haplotaxida_notID |  | Chromadorida_notID |
|  | Eunicida_notID |  | Desmodorida_notID |
|  | Boccardiella.hamata |  | Daptonema.sp..PFN.2007 |
|  | Scolelepis.sp..sco206 |  | Enoplida_notID |
| Crustacea | Nitokra.spinipes |  | Epitobrilus.stefanskii |
|  | Harpacticoida |  | Mesodorylaimus.cf..nigritulus |
|  | Chrissia.dongqianhuensis |  | Metadesmolaimus.sp..PDL.2005 |
|  | Limnocythere.inopinata |  | Monhysteridae.environmental.sample |
|  | Semicytherura.striata |  | Oncholaimidae.sp. |
|  | Podocopida |  | Triplonchida_notID |
| Gastrotricha | Chaetonotida_notID | Platyhelminthes | Bothrioplana.sinensis |
|  | Chaetonotus.cf..dispar.TK146 |  | Cirrifera.dumosa |
|  | Heterolepidoderma.loricatum |  | Dochmiotrema.limicola |
|  | Heterolepidoderma.sp..1.TK.2012 |  | Promesostoma.sp..WW.2004 |
| Mollusca | Venerida |  | Neodalyellida_notID |
|  |  |  |  |
