# Supplementary material for: Chronic trace metals effects of mine tailings on estuarine assemblages revealed by environmental DNA
Source: PeerJ. 2019 Nov 7;7:e8042. doi: 10.7717/peerj.8042 (PMC6842558; doi:10.7717/peerj.8042)
Supplement: Supplemental Information 3 — Variables were considered co-variating when the correlation coefficient (Pearson’s r) was not equal to 0 with p ≤ 0.05. Significant results (p < 0.05) are in bold. [file peerj-07-8042-s003.docx]

Table S3. Results of paired spearman correlation analysis of trace metals concentrations in sediment samples from the Rio Doce estuary in August 2017. Variables were considered co-variating when the correlation coefficient (Pearson’s r) was not equal to 0 with p ≤ 0.05. Significant results (p < 0.05) are in bold.

|  | **df** | **R** | **t** | ***p*** |
| --- | --- | --- | --- | --- |
| Fe-Zn | 42 | 0.89 | 13.25 | **< 0.0001** |
| Fe-Mn | 42 | 0.52 | 3.96 | **< 0.0001** |
| Fe-As | 42 | 0.14 | 0.96 | 0.3388 |
| Fe-Cu | 42 | 0.83 | 9.76 | **< 0.0001** |
| Fe-Pb | 42 | 0.26 | 1.79 | 0.0805 |
| Fe-Cd | 42 | 0.88 | 12.05 | **< 0.0001** |
| Fe-Co | 42 | 0.89 | 13.03 | **< 0.0001** |
| Fe-Cr | 42 | 0.88 | 12.56 | **< 0.0001** |
| Fe-Al | 42 | 0.52 | 3.98 | **0.0002** |
| Fe-Ba | 42 | -0.01 | -0.09 | 0.9233 |
