# Supplementary material for: Chronic trace metals effects of mine tailings on estuarine assemblages revealed by environmental DNA
Source: PeerJ. 2019 Nov 7;7:e8042. doi: 10.7717/peerj.8042 (PMC6842558; doi:10.7717/peerj.8042)
Supplement: Supplemental Information 4 — *Indicates significant correlations at the 0.05 level (two-tailed). [file peerj-07-8042-s004.docx]

|  | **Fe** | **Sand** | **TOM** |
| --- | --- | --- | --- |
| **eOTU richness** | *r*= 0.1090, *p*= 0.647 | *r*= 0.3254, *p*= 0.161 | *r*= -0.1116, *p*= 0.639 |
| **Fe** | 1 | *r*= -0.2342, *p=*0.320 | *r*= 0.5043, *p*= 0.023* |
| **Sand** | *r*= -0.2342,  *p*=0.320 | 1 | *r*= -0.1448,  *p*= 0.542 |
| **TOM** | *r*= 0.5043,  *p*= 0.023* | *r*= -0.1448,  *p*= 0.542 | 1 |

Table S4. Canonical correlation values (Pearson *r* and *p* values) among eOTUs and sedimentary Fe, Sand and TOM; and among co-variates (Fe, Sand and TOM). * indicates significant correlations at the 0.05 level (2-tailed).
