# Supplementary material for: Chronic trace metals effects of mine tailings on estuarine assemblages revealed by environmental DNA
Source: PeerJ. 2019 Nov 7;7:e8042. doi: 10.7717/peerj.8042 (PMC6842558; doi:10.7717/peerj.8042)
Supplement: Supplemental Information 5 [file peerj-07-8042-s005.docx]

| **Station** | **Al** | **Cr** | **Zn** | **Mn** | **Cu** | **Cd** | **Co** |
| --- | --- | --- | --- | --- | --- | --- | --- |
| ST 1 | 36544 ± 4119 | 64.5 ± 2 | 46.7 ± 4.3 | 471.6 ± 9 | 11.4 ± 0.8 | 3.8 ± 0.2 | 11.9 ± 0.1 |
| ST 2 | 29460 ± 1816 | 64.9 ± 1 | 47 ± 0.5 | 425.2 ± 2 | 14.8 ± 0.6 | 6.1 ± 0.4 | 10.3 ± 0.1 |
| ST 3 | 49973 ± 419 | 53.6 ± 3.3 | 48.7 ± 1.6 | 416.9 ± 9.2 | 15 ± 3 | 4.1 ± 0.1 | 11.4 ± 0.3 |
| ST 4 | 26881 ± 1207 | 40.8 ± 1.7 | 30 ± 0.9 | 662 ± 34.6 | 6.7 ± 1.2 | 3.1 ± 0.1 | 8.4 ± 0.6 |
| ST 5 | 38352 ± 1940 | 43.2 ± 7.8 | 26.6 ± 1.2 | 560.5 ± 57.8 | 5.2 ± 0.2 | 2.7 ± 0.2 | 7.4 ± 0.9 |
| ST 6 | 25332 ± 147 | 18 ± 0.3 | 18.3 ± 0.05 | 148.7 ± 0.3 | 4.6 ± 0.1 | 0.6 ± 0 | 3.8 ± 0 |
| ST 7 | 28660 ± 3610 | 55 ± 0.4 | 41.4 ± 0.2 | 315.1 ± 5.5 | 13.4 ± 0.7 | 3.7 ± 0.1 | 10.1 ± 0.3 |
| ST 8 | 35595 ± 3029 | 24.2 ± 3.2 | 18.2 ± 2.9 | 627.1 ± 3 | 3.3 ± 1 | 2.1 ± 0.2 | 6.5 ± 1.2 |
| ST 9 | 30108 ± 11879 | 42 ± 1 | 32.8 ± 0.2 | 503.6 ± 55.4 | 6.7 ± 0.4 | 2.9 ± 0 | 9.4 ± 0 |
| ST 10 | 38381 ± 1302 | 44.2 ± 3.9 | 39.2 ± 0.8 | 353.2 ± 4.3 | 8.3 ± 0.1 | 3.5 ± 0.1 | 9.2 ± 0.1 |
| ST 11 | 40185 ± 2618 | 54.6 ± 2.2 | 48.6 ± 1.1 | 356.3 ± 15 | 14.4 ± 0.2 | 4 ± 0.1 | 10.7 ± 0.8 |
| ST 12 | 36634 ± 11242 | 61.9 ± 16.3 | 56 ± 12.2 | 603.5 ± 128.8 | 11.8 ± 1.9 | 4.2 ± 1 | 11.3 ± 2.8 |
| ST 13 | 64597 ± 790 | 71.1 ± 8.4 | 78.1 ± 7.9 | 1002.7 ± 92.1 | 14.2 ± 1.9 | 7.1 ± 0.5 | 19.2 ± 1.8 |
| ST 14 | 38848 ± 6244 | 30.6 ± 7 | 32.3 ± 5.6 | 247.4 ± 58.2 | 7.7 ± 1.4 | 2.7 ± 0.4 | 7.5 ± 1.4 |
| ST 15 | 37060 ± 252 | 57.8 ± 0.2 | 49.4 ± 2.1 | 638.1 ± 23 | 8.8 ± 0.4 | 4.5 ± 0.3 | 13.3 ± 0.1 |
| ST 16 | 37583 ± 1771 | 32 ± 5.0 | 31.1 ± 2.4 | 318.4 ± 17.2 | 6.1 ± 0.8 | 2.9 ± 0.2 | 8.1 ± 0.2 |
| ST 17 | 9031 ± 1035 | 26.6 ± 2.4 | 20.7 ± 0.01 | 704.9 ± 10.6 | 3 ± 0.3 | 2.6 ± 0.1 | 8.1 ± 0.1 |
| ST 18 | 10714 ± 170 | 60.5 ± 2.5 | 43.4 ± 2.7 | 981.5 ± 60.6 | 11.5 ± 0.7 | 4.2 ± 0.2 | 11.3 ± 0.2 |
| ST 19 | 29767 ± 2616 | 41.9 ± 1.9 | 31.5 ± 0.6 | 561.9 ± 17.4 | 9.7 ± 1.1 | 3.2 ± 0.1 | 8.8 ± 0.6 |
| ST 20 | 28972 ± 437 | 19 ± 0.2 | 20.7 ± 0.7 | 140.8 ± 3 | 6.4 ± 0.2 | 2.5 ± 0.1 | 4.3 ± 0.1 |
| ST 22 | 40131 ± 1175 | 30.6 ± 1.7 | 32.3 ± 3.7 | 196.7 ± 8.5 | 8.1 ± 1.1 | 2.1 ± 1.3 | 7.5 ± 0.4 |
| ST 23 | 45111 ± 2561 | 57.3 ± 1.9 | 44.8 ± 2.9 | 553.5 ± 14.4 | 10.2 ± 0.7 | 3.9 ± 0.3 | 12.8 ± 0.6 |

Table S4 Trace metal concentrations (mg.kg^-1^) in sediments at each sampling site of the Rio Doce estuary in August 2017.
